# Multi-omics integration and clinical validation identify CKAP2 as a diagnostic biomarker for bladder cancer

**DOI:** 10.1186/s12957-026-04464-7

**Published:** 2026-06-23

**Authors:** Xinxin Li, Jinshan Yang, Jiazi Cha, Hao Xie, Changming Dong, Yufan Yang, Yue Li, Jiahao Guo, Chunhua Lin

**Affiliations:** 1 School of Clinical Medicine, Shandong Second Medical University, Weifang, 261053 China; 2 Department of Urology, The Affiliated Yantai Yuhuangding Hospital of Qingdao University, Yantai, China

**Keywords:** Bladder cancer, CKAP2, Diagnostic biomarker, Logistic regression, Multi-omics, Mendelian randomization

## Abstract

**Background:**

Bladder cancer (BC) remains a prevalent malignancy with a high recurrence rate; however, the lack of specific biomarkers hinders early detection and effective management. Although Cytoskeleton-Associated Protein 2 (CKAP2) has been implicated in tumorigenesis, its diagnostic value and biological function in BC have not been fully elucidated. This study aimed to identify and rigorously validate CKAP2 as an important diagnostic biomarker using a multi-omics approach integrated with clinical validation.

**Methods:**

Differential expression analysis and machine learning-based feature selection were employed to screen for candidate biomarkers, identifying CKAP2 as a key gene of interest. Mendelian randomization (MR) was used to assess the causal relationship between CKAP2 and BC risk. To evaluate clinical utility, a diagnostic model based on logistic regression was established and validated in an independent real-world cohort of 127 tissue samples, including 50 pairs of matched tumor and adjacent normal tissues, 21 independent tumor tissues, and 6 independent adjacent normal tissues. Additionally, single-cell RNA sequencing (scRNA-seq) and cell–cell communication analyses were performed to dissect the role of CKAP2 within the tumor microenvironment.

**Results:**

CKAP2 expression was significantly upregulated in BC tissues compared to that in normal tissues (log2FoldChange = 1.53, p < 10⁻^5^). MR analysis provided evidence supporting a potential causal association between elevated CKAP2 expression and increased BC risk. In the external clinical validation, the CKAP2-based model demonstrated exceptional discrimination with an Area Under the Curve (AUC) of 0.931. Calibration curves indicated a high agreement between the predicted and observed probabilities, while Decision Curve Analysis (DCA) confirmed a substantial clinical net benefit. Mechanistically, scRNA-seq localized high CKAP2 expression primarily to cancer cells, and cell–cell communication analysis suggested that CKAP2 may play an important role in modulating tumor-stroma interactions, particularly with fibroblasts.

**Conclusion:**

Our integrated analysis supports the potential utility of CKAP2 as a diagnostic biomarker for BC. By integrating multi-omics analysis, causal inference, and validation in an independent clinical cohort, these results consistently point to CKAP2’s diagnostic potential and suggest its involvement in BC progression.

**Supplementary Information:**

The online version contains supplementary material available at https://doi.org/10.1186/s12957-026-04464-7.

## Introduction

Bladder cancer (BC) represents a significant and growing global health burden [1]. Globally, the age-standardized incidence rate of BC increased from 1992 to 2021, and projections indicate that this upward trend will continue in specific regions through 2046 [1]. Accompanying this epidemiological shift is a widening inequality in BC burden across countries with differing socio-demographic indices (SDI). Furthermore, the disability-adjusted life-years (DALYs) rate for BC rises steadily with age and is consistently higher in men than in women across all age groups, underscoring the disproportionate impact of BC on older male populations [1]. The disease is characterized by a high propensity for recurrence, progression, and metastasis, underscoring the critical need for early detection to improve patient survival rates.

Despite advances in medical technology, current diagnostic modalities face substantial limitations. Conventional tools such as computed tomography (CT) and white-light cystoscopy (WLC) are limited in sensitivity, specificity, and tumor characterization, particularly for small or flat lesions, highlighting the need for innovative, non-anatomical approaches [2]. While cystoscopy remains the gold standard for BC diagnosis, its performance in detecting carcinoma in situ and small or flat lesions is suboptimal, which can contribute to incomplete resections and underdiagnosis [2]. Urinary cytology often lacks sufficient sensitivity for low-grade tumors, further limiting its reliability as a standalone diagnostic tool [3]. Recent advances in management have introduced non-invasive diagnostic strategies, including liquid biopsy approaches that analyze circulating tumor DNA (ctDNA) and gene expression signatures, offering new avenues for patient stratification and monitoring [4]. Concurrently, therapeutic strategies have expanded beyond platinum-based chemotherapy: immune checkpoint inhibitors (ICIs) targeting the PD-1/PD-L1 axis have become essential options for patients with advanced disease or those ineligible for cisplatin, and antibody–drug conjugates (ADCs) have demonstrated improved clinical outcomes in metastatic urothelial carcinoma [5]. However, these emerging diagnostics and therapeutics have their own limitations; many novel approaches require further validation, and their integration into routine clinical practice remains challenging. Consequently, there is an urgent imperative to identify novel, non-invasive, and highly specific biomarkers that can complement these emerging strategies to facilitate early diagnosis and optimize clinical management.

The cell cycle is a fundamental process in tumorigenesis, and its dysregulation is a hallmark of malignancy [6]. Cytoskeleton-Associated Protein 2 (CKAP2), a microtubule-associated protein essential for mitotic spindle formation and cell cycle regulation, has been implicated in the progression of various cancers [7]. However, the specific diagnostic potential and biological function of CKAP2 in the context of BC remain largely unexplored.

While recent advancements in high-throughput sequencing and multi-omics approaches have accelerated biomarker discovery, the translation of these findings into clinical practice remains challenging. A major limitation in current BC research is the reliance on public databases without robust validation in independent clinical cohorts, leading to a gap between bioinformatic prediction and real-world utility [8]. Furthermore, most studies focus on correlations rather than causations. Mendelian randomization (MR), which leverages genetic variants as instrumental variables, offers a powerful method for inferring causal relationships between exposures and disease outcomes [9]. Yet, the application of MR to elucidate the causal role of specific biomarkers in BC has been limited. To date, a comprehensive study integrating multi-omics mining, causal inference, and rigorous clinical validation to establish CKAP2 as a reliable diagnostic marker for BC has not been conducted.

To address these gaps, this study employed an integrated strategy combining RNA sequencing (RNA-seq), single-cell RNA sequencing (scRNA-seq), machine learning (ML), and Mendelian randomization (MR) to systematically characterize the role of CKAP2 in BC. We first utilized differential expression analysis and Weighted Gene Co-expression Network Analysis (WGCNA) on both in-house and TCGA datasets to screen for BC-related gene modules, followed by LASSO regression to identify robust candidate features. Unlike previous studies, we incorporated MR analysis to provide genetic evidence supporting a causal link between CKAP2 and BC risk. Mechanistically, scRNA-seq and cell–cell communication analyses were performed to dissect the interplay between CKAP2-expressing cancer cells and the tumor microenvironment (TME), particularly the fibroblasts. Most importantly, to ensure clinical applicability, we validated CKAP2 expression in an independent real-world cohort of 127 tissue samples, including 50 pairs of matched tumor and adjacent normal tissues, 21 independent tumor tissues, and 6 independent adjacent normal tissues. and developed a diagnostic model based on the results. By evaluating the model's performance using ROC, calibration, and decision curve analyses (DCA), we provide solid evidence supporting CKAP2 as an important and clinically actionable diagnostic biomarker for bladder cancer.

## Materials and methods

### Study design

This study employed a multi-stage, multi-omics strategy to identify and validate Cytoskeleton-Associated Protein 2 (CKAP2) as an important diagnostic biomarker for bladder cancer (BC). The research framework integrated bulk RNA sequencing (RNA-seq), single-cell RNA sequencing (scRNA-seq), machine learning algorithms, and Mendelian randomization (MR) to ensure comprehensive biomarker characterization. Initially, differential expression analysis was conducted on both an in-house BC cohort and the TCGA-BLCA dataset to screen for dysregulated genes. Weighted Gene Co-expression Network Analysis (WGCNA) was then applied to identify gene modules significantly correlated with BC progression. To refine candidate biomarkers, Least Absolute Shrinkage and Selection Operator (LASSO) regression was utilized. Subsequently, the causal relationship between CKAP2 and BC risk was assessed using MR analysis with genetic variants as instrumental variables. CKAP2 expression patterns were validated across bulk and single-cell transcriptomic datasets. To explore biological mechanisms, functional enrichment analyses (GO and KEGG) and cell–cell communication analysis (CellChat) were performed. Finally, to establish clinical utility, a diagnostic model was developed and validated in an independent clinical cohort of 127 tissue samples.

### Data collection and processing

#### Public datasets

Gene expression data and corresponding clinical information for BC were retrieved from The Cancer Genome Atlas (TCGA-BLCA) via the Genomic Data Commons (GDC) portal. This dataset included RNA-seq profiles from both tumor and adjacent normal tissues [10]. Raw counts were pre-processed and normalized to correct for technical variations and ensure cross-sample comparability. Single-cell RNA-seq data were obtained from the Gene Expression Omnibus (GEO) under accession number GSE145137, comprising samples from a BC patient and patient-derived xenografts (PDX) [11].

#### In-house clinical cohort

To validate bioinformatic findings, an independent in-house cohort was established. Tissue samples were collected from 127 tissue samples (including 71 bladder cancer tissues and 56 adjacent normal tissues) at The Affiliated Yantai Yuhuangding Hospital of Qingdao University. The study protocol was approved by the institutional ethics committee. Total RNA was extracted and libraries were constructed using the MGIEasy RNA Library Prep Kit [12]. Sequencing was performed on the DNBSEQ-T1&T5 platform (BGI-Shenzhen) to generate high-quality transcriptomic data.

### Single-cell RNA-seq analysis

Raw scRNA-seq data underwent rigorous quality control. Cells exhibiting low sequencing quality, high mitochondrial gene content, or mixed human-mouse signals (in PDX samples) were excluded based on nFeature and nCount thresholds [13]. Post-filtering, data normalization, variable feature identification, and scaling were performed using the Seurat package [14]. Dimensionality reduction was achieved via Principal Component Analysis (PCA) and Uniform Manifold Approximation and Projection (UMAP) [15]. Cell clusters were identified using the K-Nearest Neighbor (KNN) algorithm and annotated based on canonical marker genes to distinct cell types [16].

### Identification of candidate biomarkers

#### WGCNA analysis

Weighted Gene Co-expression Network Analysis (WGCNA) was utilized to construct a scale-free co-expression network from bulk RNA-seq data [17]. An optimal soft-thresholding power was selected to ensure network connectivity. Hierarchical clustering identified gene modules, which were then correlated with clinical traits to pinpoint modules significantly associated with BC progression.

#### LASSO regression

To identify robust diagnostic features, the intersection of DEGs from TCGA, the in-house cohort, and key WGCNA module genes was analyzed. Least Absolute Shrinkage and Selection Operator (LASSO) regression was performed using the glmnet R package [18]. Ten-fold cross-validation determined the optimal regularization parameter (λ) to minimize binomial deviance [19]. Genes retaining non-zero coefficients were selected as candidate biomarkers for further investigation.

### Mendelian Randomization (MR) analysis

To infer causality between candidate genes and BC risk, two-sample MR analysis was conducted. Genetic instruments (expression quantitative trait loci, eQTLs) for the exposure genes were sourced from the IEU OpenGWAS project (https://gwas.mrcieu.ac.uk/), with the underlying data generated by the eQTLGen Consortium. The eQTLGen Consortium dataset is derived from whole blood samples of 31,684 individuals and is designed to investigate the genetic regulation of blood gene expression [20]. While outcome data for BC were obtained from the FinnGen consortium [21]. Instruments were filtered for genome-wide significance (p < 5e-8) and independence (r^2^ < 0.001, window > 10,000 kb) [22]. Causal estimates were generated using the Inverse Variance Weighted (IVW) method, supplemented by MR-Egger and Weighted Median methods to assess robustness and pleiotropy.

### Functional enrichment and immune landscape analysis

Biological functions of CKAP2-correlated genes were explored using Gene Ontology (GO) and Kyoto Encyclopedia of Genes and Genomes (KEGG) pathway analyses via the clusterProfiler package [23]. To characterize the tumor immune microenvironment (TIME), single-sample Gene Set Enrichment Analysis (ssGSEA) was performed. Immune cell infiltration scores were calculated, and differential abundance between tumor and normal tissues was assessed using the Wilcoxon test. Correlation analyses linked CKAP2 expression with immune cell infiltration levels to reveal potential immunomodulatory roles.

### Analysis of mismatch repair gene expression

To evaluate microsatellite instability (MSI)-related indicators as suggested by Nádorvári et al. [24], we examined the expression levels of core mismatch repair (MMR) genes (MLH1, MSH2, MSH6, and PMS2) in our internal RNA-seq cohort [25]. Samples were stratified into CKAP2-high and CKAP2-low groups based on the median CKAP2 expression value. Differences in MMR gene expression between the two groups were assessed using the Wilcoxon rank-sum test. Spearman's rank correlation was employed to evaluate the association between CKAP2 and individual MMR gene expression levels. All statistical analyses were performed in R (version 4.3.0), and a two-sided P < 0.05 was considered statistically significant.

### Cell–cell communication analysis

Intercellular communication within the TME was inferred using the CellChat R package based on scRNA-seq data [26]. Ligand-receptor interactions were identified, and communication probabilities were computed between CKAP2-expressing cancer cells and other stromal components, particularly fibroblasts. Circle plots were generated to visualize the strength and number of significant interactions, elucidating the role of CKAP2 in mediating tumor-stroma crosstalk.

### Patient enrollment and diagnostic model construction

To facilitate clinical translation, the diagnostic utility of CKAP2 was validated in an independent in-house cohort. Tissue samples were collected from 127 tissue samples, and CKAP2 expression levels were quantified. Continuous variables were assessed for normality using the Shapiro–Wilk test and compared using the Mann–Whitney U test [27].

A univariate logistic regression model was established to evaluate the predictive value of CKAP2 for bladder cancer. The association between CKAP2 expression and disease risk was quantified using odds ratios (OR) with 95% confidence intervals (CI). To visualize the continuous relationship between biomarker levels and tumor probability, a predicted probability curve was plotted. The discriminative performance of the model was assessed via receiver operating characteristic (ROC) curve analysis, calculating the area under the curve (AUC) and its 95% CI. The optimal cutoff value was determined using the Youden index. Model calibration was evaluated using a calibration curve with 1,000 bootstrap resamples to assess the agreement between predicted and observed probabilities [28]. Furthermore, Decision Curve Analysis (DCA) was employed to quantify the clinical net benefit of the model across a range of threshold probabilities. All statistical analyses were performed using R software, and a two-sided P-value < 0.05 was considered statistically significant.

### Tissue specimens and immunohistochemistry

Paraffin-embedded Sects. (6 cases) were deparaffinized, rehydrated, and subjected to antigen retrieval in citrate buffer by microwave heating for 30 min. Endogenous peroxidase was blocked with 3% H₂O₂‑methanol for 10 min. After blocking with 3% BSA, sections were incubated with rabbit anti‑CKAP2 antibody (1:200, 25,486–1-AP, Proteintech) overnight at 4°C. Following PBS washes, sections were incubated with a goat anti‑rabbit polymer (KIT-5005, Maixin) for 20 min. Signals were visualized using DAB, and sections were counterstained with hematoxylin. Whole‑slide images were captured with a digital slide scanner (VS200, Olympus).

### Cell culture and siRNA transfection

The human bladder cancer cell line T24 was cultured in RPMI-1640 medium supplemented with 10% fetal bovine serum (FBS) at 37°C in a 5% CO₂ atmosphere. Small interfering RNA targeting CKAP2 (si-CKAP2) and a negative control siRNA (si-NC) were synthesized by GenePharma (Qingdao, China). T24 cells were seeded in 6-well plates at a density of 2 × 10^5^ cells/well and allowed to attach overnight. Transfection was performed using Lipofectamine 3000 (Invitrogen) according to the manufacturer’s protocol. At 48 h post-transfection, knockdown efficiency was verified by quantitative real-time PCR and Western blotting. T24 cells transfected with si-CKAP2 or si-NC were used for subsequent functional assays.

### Wound healing assay

T24 cells were seeded into 6-well plates at a density of 5 × 10^5^ cells/well and cultured until reaching approximately 90% confluence. The monolayer was scratched using a sterile 200-μL pipette tip to create a linear wound. Detached cells were removed by washing with PBS, and the medium was replaced with serum-free RPMI-1640 to minimize proliferation [29]. Images of the wound area were captured immediately (0 h) and after 24 h using an inverted microscope.

### Transwell migration and invasion assays

Transwell migration and invasion assays were performed using commercial chambers according to the manufacturer’s instructions. For the migration assay, 2 × 10^4^ transfected T24 cells in 200 μL serum-free medium were seeded into the upper chamber of an 8.0-μm pore polycarbonate membrane insert [30]. The lower chamber was filled with 500 μL medium containing 10% FBS as a chemoattractant. After incubation at 37°C for 24 h, cells that had migrated to the lower surface of the membrane were fixed with 4% paraformaldehyde and stained with 0.1% crystal violet [30]. For the invasion assay, inserts pre-coated with Matrigel were used. T24 cells (5 × 10^4^ per well) were seeded into the upper chamber in serum-free medium, and the subsequent steps were identical to those of the migration assay [30]. After staining, cells in five randomly selected fields were counted under a light microscope. All experiments were performed in triplicate.

## Results

### Differential gene expression and co-expression network

**Fig. 1:**
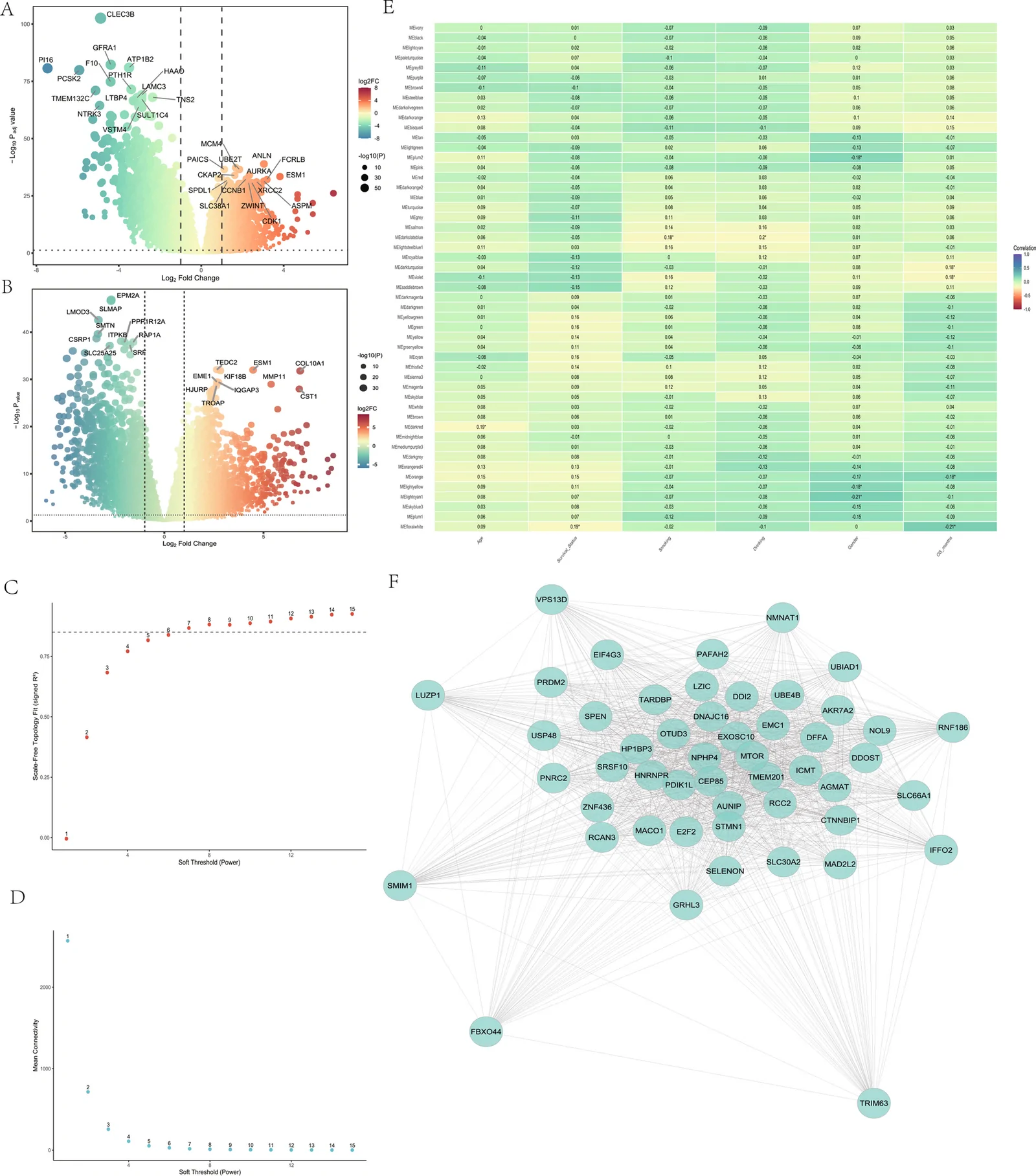
Differential gene expression analysis and WGCNA in bladder cancer. **A** Volcano plot of differentially expressed genes (DEGs) based on the self-generated dataset, highlighting significantly upregulated and downregulated genes. **B** Volcano plot of DEGs from TCGA bladder cancer data, illustrating expression changes between tumor and normal samples. **C**–**E** Weighted gene co-expression network analysis (WGCNA) based on the self-generated dataset, including soft threshold selection, module identification, and module-trait correlation analysis. **F** Heatmap of the top 50 genes in the MEturquoise module, which showed the strongest association with clinical traits, suggesting potential hub genes for further investigation

To elucidate the molecular landscape of bladder cancer (BC), we performed differential expression analysis on both our in-house RNA-seq cohort and the TCGA-BLCA dataset. Volcano plots revealed distinct gene expression profiles, identifying a substantial number of significantly up- and downregulated genes in tumor tissues compared to adjacent normal controls in both the in-house (Fig. 1A) and TCGA datasets (Fig. 1B). To further capture gene modules associated with tumorigenesis, Weighted Gene Co-expression Network Analysis (WGCNA) was applied to the in-house dataset. A soft-thresholding power of β = 7 was selected to construct a scale-free network (Fig. 1C, D). Among the identified modules, the MEturquoise module exhibited the strongest positive correlation with BC clinical traits and was thus selected for downstream analysis (Fig. 1E). The top 50 hub genes within this module were visualized in a protein–protein interaction network (Fig. 1F), highlighting key regulatory candidates such as STMN1, a regulator of microtubule dynamics, and SPEN, a modulator of Notch signaling, both of which are implicated in cancer progression.

### Functional annotation and immune microenvironment characterization

**Fig. 2:**
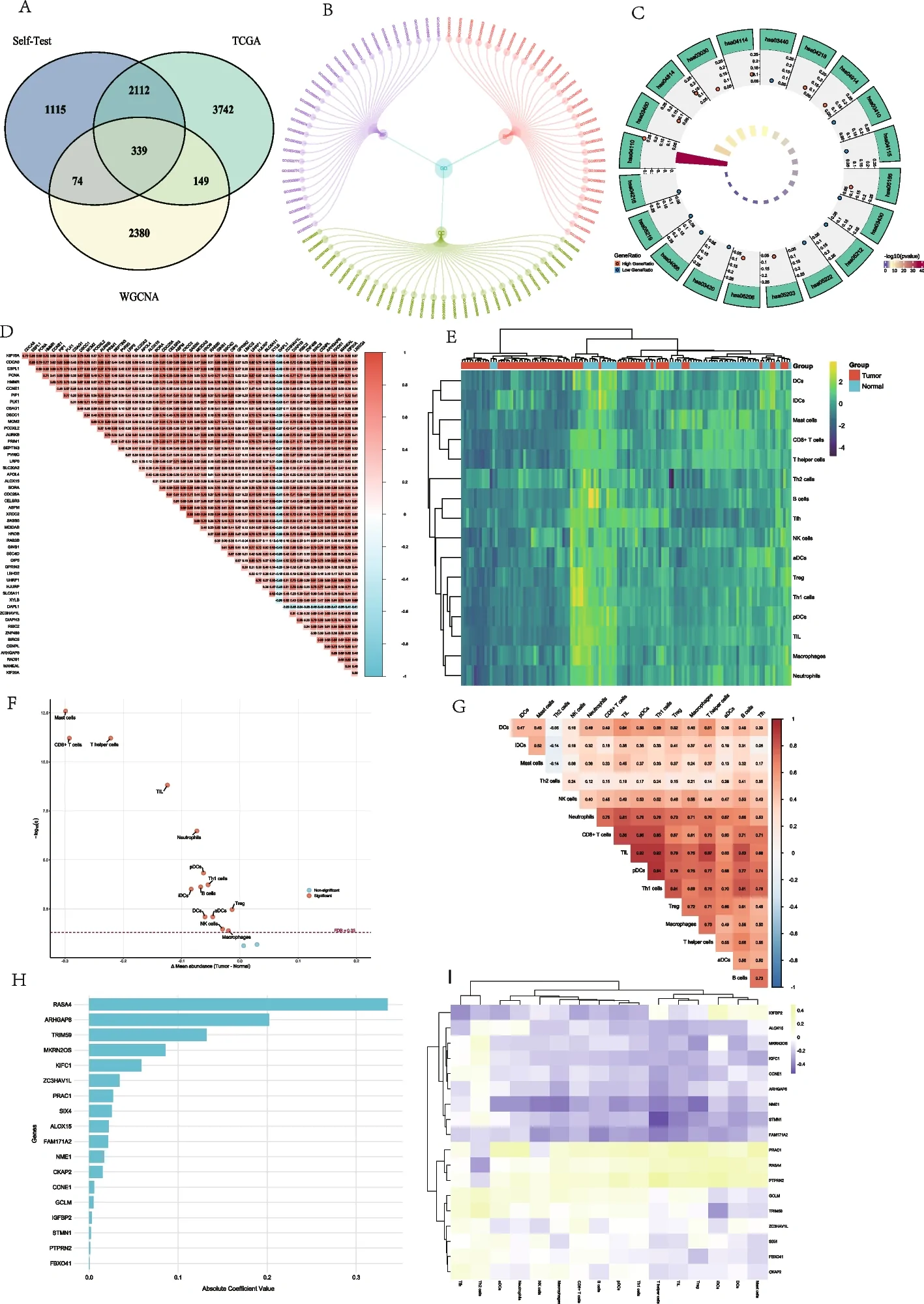
Identification, functional annotation, and immune infiltration analysis of intersecting genes. **A** Venn diagram showing the intersection of differentially expressed genes (DEGs) from the self-generated dataset, TCGA bladder cancer dataset, and genes in the MEturquoise module identified by WGCNA. **B** Gene Ontology (GO) enrichment analysis of the intersecting genes. **C** KEGG pathway analysis of the intersecting genes. **D** Correlation analysis of the intersecting genes. **E** ssGSEA-based immune cell distribution in tumor and adjacent normal tissues, with red and blue indicating tumor and normal tissues, respectively. **F** Relative infiltration abundance of immune cell types across samples. **G** Correlation analysis among different immune cell populations. **H** LASSO regression analysis identifying key genes associated with bladder cancer progression. **I** Correlation analysis between LASSO-selected bladder cancer-related genes and immune cell types

We identified 339 overlapping genes from the intersection of differentially expressed genes (DEGs) in the in-house and TCGA datasets with the MEturquoise WGCNA module (Fig. 2A). Functional enrichment analysis revealed that these genes were significantly enriched in biological processes critical for cell proliferation, including chromosome segregation, mitotic nuclear division, and DNA replication (Fig. 2B). Molecular function analysis further implicated tubulin binding and ATP-dependent DNA helicase activity, pointing to dysregulated microtubule dynamics and DNA repair mechanisms. Consistent with these findings, KEGG pathway analysis highlighted the cell cycle, DNA replication, and homologous recombination as the most enriched pathways (Fig. 2C), reinforcing the role of these genes in driving genomic instability and uncontrolled proliferation.

Beyond molecular pathways, we characterized the immune landscape of BC using ssGSEA. Tumor tissues exhibited a generally suppressed immune profile compared to normal tissues, with significantly reduced infiltration of cytotoxic immune cells such as CD8 + T cells and macrophages (Fig. 2E, F). Correlation analysis revealed complex interactions among immune cell subsets; notably, immunosuppressive populations like regulatory T cells (Tregs) showed strong correlations with other stromal components (Fig. 2G). To pinpoint prognostic biomarkers, LASSO regression was applied to the intersecting gene set, identifying 18 key candidates, including RASA4, ARHGAP8, and TRIM59 (Fig. 2H). Correlation analysis between these markers and immune infiltration scores demonstrated that genes such as STMN1 and ARHGAP8 were negatively correlated with cytotoxic lymphocytes (NK cells, CD8 + T cells) (Fig. 2I).

### Causal validation of candidate genes via mendelian randomization

**Fig. 3:**
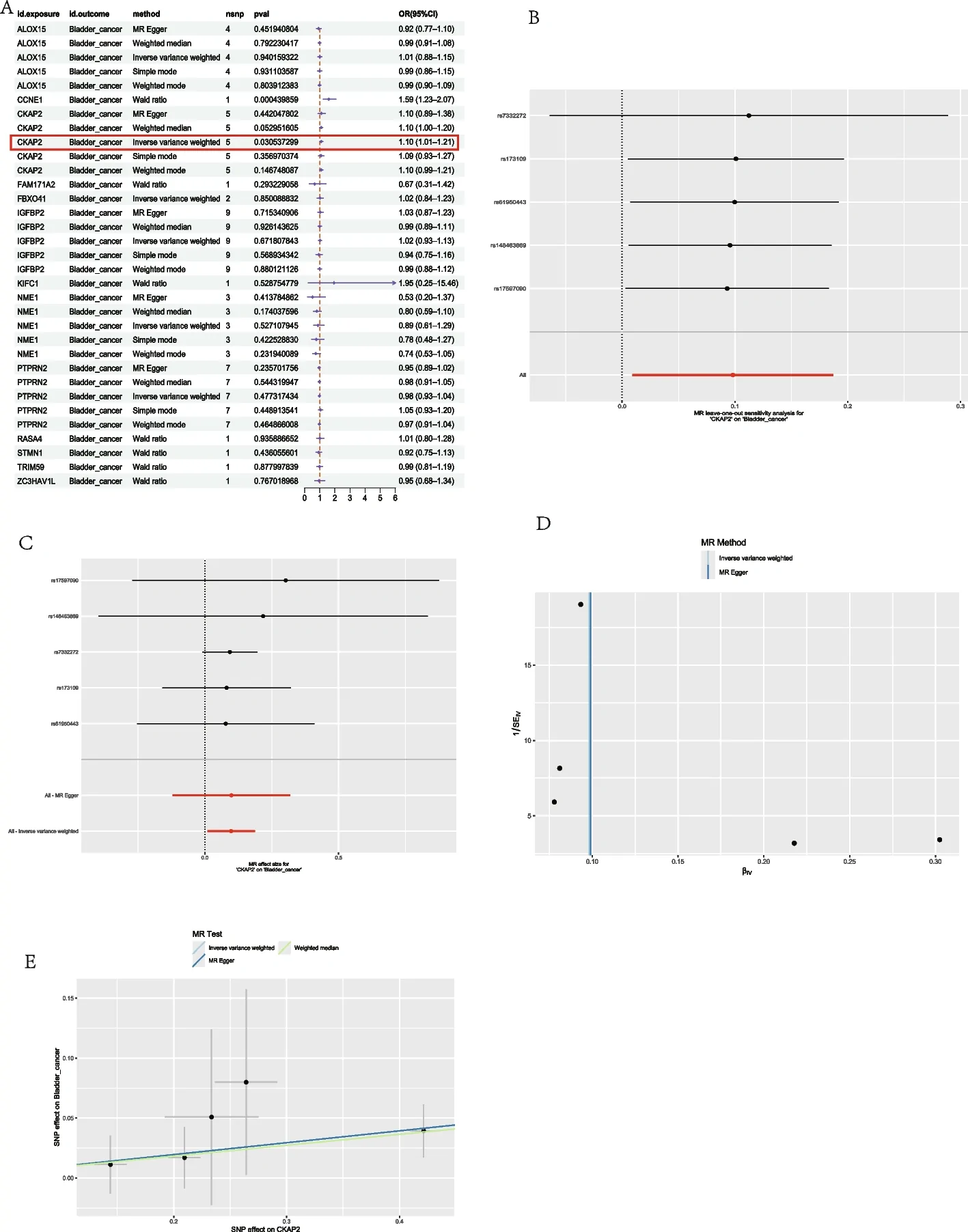
Mendelian randomization (MR) analysis of CKAP2 and its association with bladder cancer. **A** Forest plot of MR model results showing the estimated effects of CKAP2 on bladder cancer risk across different instrumental variables (SNPs). **B** Leave-one-out sensitivity analysis for CKAP2, with the red line representing the pooled effect after sequentially excluding each SNP, indicating the robustness of the MR estimates. **C** Forest plot of MR effect estimates for individual SNPs related to CKAP2, with the red line denoting the overall effect estimate, supporting CKAP2 as a potential risk factor. **D** Funnel plot assessing the presence of directional pleiotropy among the three selected SNPs used in MR analysis. **E** Scatter plot of MR results across three models, where each point represents a single instrumental variable (IV); the x-axis indicates SNP effects on exposure, and the y-axis shows their effects on outcome. Error bars represent the 95% confidence intervals

To discern causality from correlation, we performed Two-Sample Mendelian Randomization (MR) on the LASSO-selected candidates. Of the 18 initial genes, 13 had sufficient valid instrumental variables (eQTLs) in the GWAS datasets (Fig. 3A). Among these, CKAP2 emerged as the most significant causal risk factor for BC. The primary Inverse Variance Weighted (IVW) analysis yielded an odds ratio (OR) of 1.103 (95% CI: 1.009–1.206, p = 0.0305), indicating that genetically predicted upregulation of CKAP2 is causally associated with increased BC risk. Sensitivity analyses confirmed the robustness of this finding: the leave-one-out analysis demonstrated that the causal estimate was not driven by any single SNP (Fig. 3B), and the MR-Egger regression showed no evidence of significant horizontal pleiotropy (Fig. 3E). While other genes showed varying degrees of association, CKAP2 provided the most consistent evidence across different MR methods (Fig. 3C, D), positioning it as a prioritized candidate for further validation.

### Expression patterns and clinical relevance of CKAP2

**Fig. 4:**
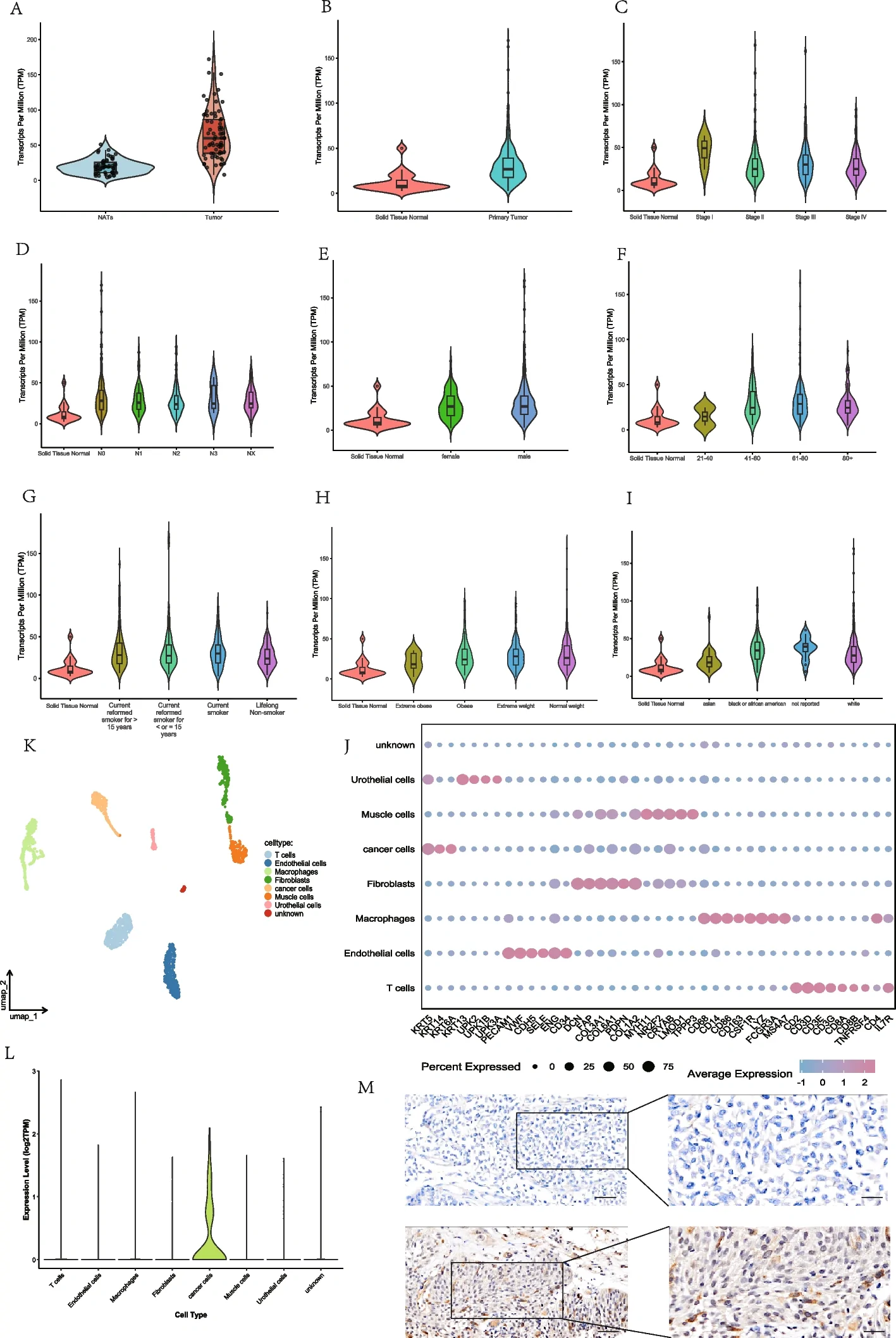
Expression patterns of CKAP2. **A** Violin plot showing the expression levels of CKAP2 in tumor versus adjacent normal tissues based on the self-generated dataset. **B**–**I** Expression analysis of CKAP2 in the TCGA bladder cancer dataset. **B** Comparison of CKAP2 expression between tumor and normal samples. **C** Expression levels stratified by pathological stage. **D** Expression levels based on lymph node metastasis status. **E** Gender-based comparison of CKAP2 expression in normal and tumor tissues. **F** Age group–specific distribution of CKAP2 expression. **G** Expression differences associated with smoking history. **H** Expression patterns across body mass index (BMI) categories. **I** Racial/ethnic subgroup analysis of CKAP2 expression in normal and tumor tissues. **J** Proportion of marked gene expression across different cell types. **K** Distribution of cell clusters in bladder cancer. **L** Expression intensity of CKAP2 across identified cell clusters. **M** Representative CKAP2 immunohistochemical images in bladder cancer and adjacent non-tumor tissues (n = 6). Left panel (scale bar = 100 μm). Right panel (scale bar = 50 μm)

We extensively validated CKAP2 expression patterns across multiple datasets. In our in-house RNA-seq cohort, CKAP2 was markedly upregulated in tumor tissues compared to adjacent normal tissues (p < 2.2 × 10–16) (Fig. 4A; Fig S1), a finding robustly corroborated by the TCGA-BLCA cohort (Fig. 4B). To resolve cellular heterogeneity, we analyzed scRNA-seq data (GSE145137), identifying eight major cell clusters (Fig. 4K). Notably, CKAP2 expression was predominantly localized to the cancer cell population rather than stromal or immune cells (Fig. 4L), supporting its role as a tumor-intrinsic driver. Immunohistochemistry also indicates that CKAP2 is highly expressed in cancer tissues (Fig. 4M). Clinically, elevated CKAP2 levels correlated with aggressive disease features. Expression was significantly higher in non-muscle-invasive BC (NMIBC) compared to muscle-invasive BC (MIBC) (Fig. 4C) and remained elevated across all lymph node metastasis stages (Fig. 4G). Interestingly, we observed that CKAP2 expression decreases with advancing tumor stage in bladder cancer (Fig. 4C). Stratification by demographics showed consistently high expression in tumors regardless of gender (Fig. 4D) or age, with a pronounced elevation in patients aged 41–80 (Fig. 4E).

### Molecular mechanisms and microenvironmental interactions

**Fig. 5:**
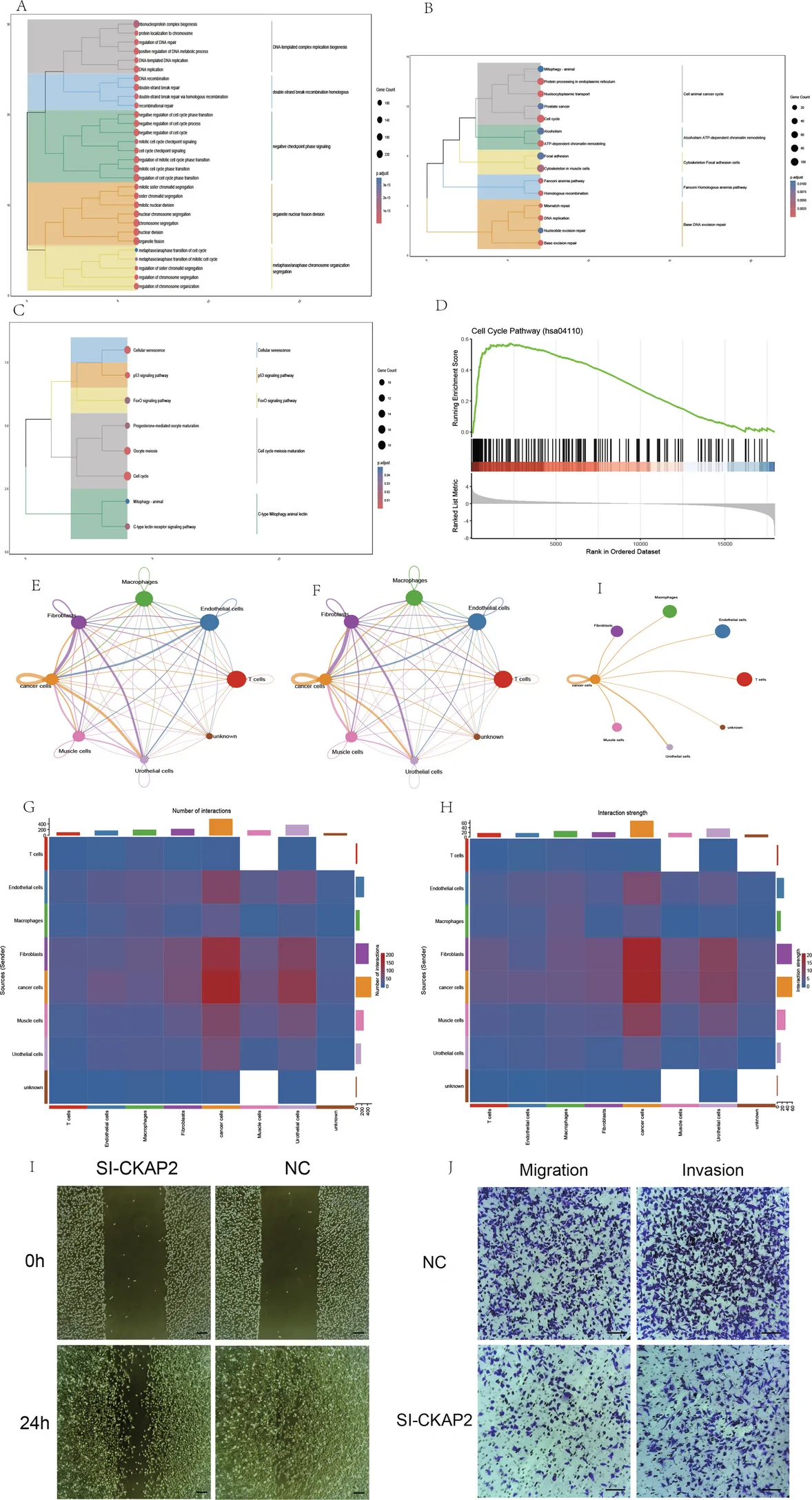
Functional enrichment and cell–cell communication analysis of CKAP2-related genes. **A** GO enrichment analysis of CKAP2-associated genes identified from bulk RNA-seq data. **B** KEGG pathway enrichment of CKAP2-correlated genes from bulk transcriptomic data. **C** KEGG pathway enrichment of CKAP2-related genes derived from single-cell transcriptomic data. **D** GSEA results showing enrichment of CKAP2-related genes in the Cell Cycle pathway. **E** Number of inferred cell–cell interactions estimated by CellChat analysis across all cell types in bladder cancer. **F** Overall interaction strength between cell types based on ligand–receptor pairing. **G** Heatmap showing the number of interactions between cancer cells and other cell populations. **H** Heatmap displaying the interaction strength among cell types. **I** Network diagram showing interactions between tumor cells and multiple surrounding cell types in the tumor microenvironment. **J** Wound healing assay of T24 cells in si-CKAP1 and NC-CKAP2 groups at 0 and 24 h (Scale bar = 100 μm). **K** migration and invasion assays of T24 cells in si-CKAP1 and NC-CKAP2 groups (Scale bar = 100 μm)

Functional enrichment analyses elucidated the biological machinery correlated with CKAP2 expression. GO analysis indicated that CKAP2-correlated genes are integral to chromosome segregation and cell cycle phase transitions (Fig. 5A). KEGG pathways consistently highlighted the cell cycle, DNA replication, and base excision repair (Fig. 5B). GO enrichment analysis revealed a high degree of similarity between the CKAP2-high groups of the internal cohort and the TCGA cohort, with both consistently highlighting processes such as chromosome segregation, nuclear division, and nuclear chromosome segregation. In contrast, no significant enrichment was observed in the CKAP2-low groups (Figure S2). The CKAP2-high subgroups from the internal cohort and TCGA datasets exhibited highly concordant enrichment of the Cell cycle pathway in KEGG analysis (Figure S3). scRNA-seq analysis comparing CKAP2-high versus CKAP2-low cancer cells confirmed that high expression is associated with the activation of cell cycle and DNA repair pathways (Fig. 5C), a result further validated by GSEA in bulk tissues (Fig. 5D). In line with these findings, functional assays confirmed that CKAP2 promoted the proliferation and migration of bladder cancer cells (Fig. 5J, K). Notably, all four MMR genes exhibited significantly higher expression in the CKAP2-high group compared with the CKAP2-low group (Figure S4; *P* = 0.0048 for *MLH1*, *P* = 1.88 × 10^–15^ for *MSH2*, *P* = 9.85 × 10^⁻10^ for *MSH6*, and *P* = 1.11 × 10⁻^9^ for *PMS2*). Spearman correlation analysis further confirmed positive correlations between CKAP2 and each MMR gene (*P* < 0.05). Investigating the tumor microenvironment via CellChat revealed potential intercellular communication patterns involving CKAP2-expressing cancer cells (Fig. 5E, F). These cells exhibited inferred ligand-receptor interactions with fibroblasts and macrophages (Fig. 5G-I), suggesting a possible role for CKAP2 in tumor-stroma crosstalk that warrants further experimental investigation.

### Clinical validation and diagnostic model construction

We validated the diagnostic potential of CKAP2 in an independent clinical cohort of 127 tissue samples, including 50 pairs of matched tumor and adjacent normal tissues, 21 independent tumor tissues, and 6 independent adjacent normal tissues. Analysis of baseline expression profiles revealed a distinct disparity between the two groups: the median CKAP2 expression was markedly higher in tumor tissues compared to normal tissues (Table 1).

**Table 1:** Baseline clinicopathological characteristics of 127 tissue samples

Characteristics	Paired Patients (N = 50)	Independent Cancer (N = 21)	Independent Normal (N = 6)	P-value
Demographics				
Age (years), mean ± SD	68.30 ± 8.57	67.57 ± 10.24	58.83 ± 8.68	0.052
Gender (Male), n (%)	41 (82.0%)	18 (85.7%)	5 (83.3%)	0.932
Lifestyle				
Smoking history (Yes), n (%)	18 (36.0%)	11 (52.4%)	2 (33.3%)	0.407
Drinking history (Yes), n (%)	14 (28.0%)	6 (28.6%)	1 (16.7%)	0.817
Clinical Outcomes				
Death, n (%)	29 (58.0%)	9 (42.9%)	1 (16.7%)	0.108
OS (months), mean ± SD	41.69 ± 22.85	39.92 ± 23.27	58.09 ± 15.10	0.201
Pathological Features				
Grade (High), n (%)	42 (84.0%)	19 (90.5%)	-	0.697
T-category (T2-T4), n (%)	37 (74.0%)	17 (81.0%)	-	0.76
Muscle-invasive (Yes), n (%)	37 (74.0%)	17 (81.0%)	-	0.76
Biomarker Expression				
CKAP2 (TPM) (Cancer tissue)	69.14 ± 34.62	55.99 ± 29.37	-	0.123
CKAP2 (TPM) (Normal tissue)	19.27 ± 10.87	-	25.02 ± 10.99	0.225

**Fig. 6:**
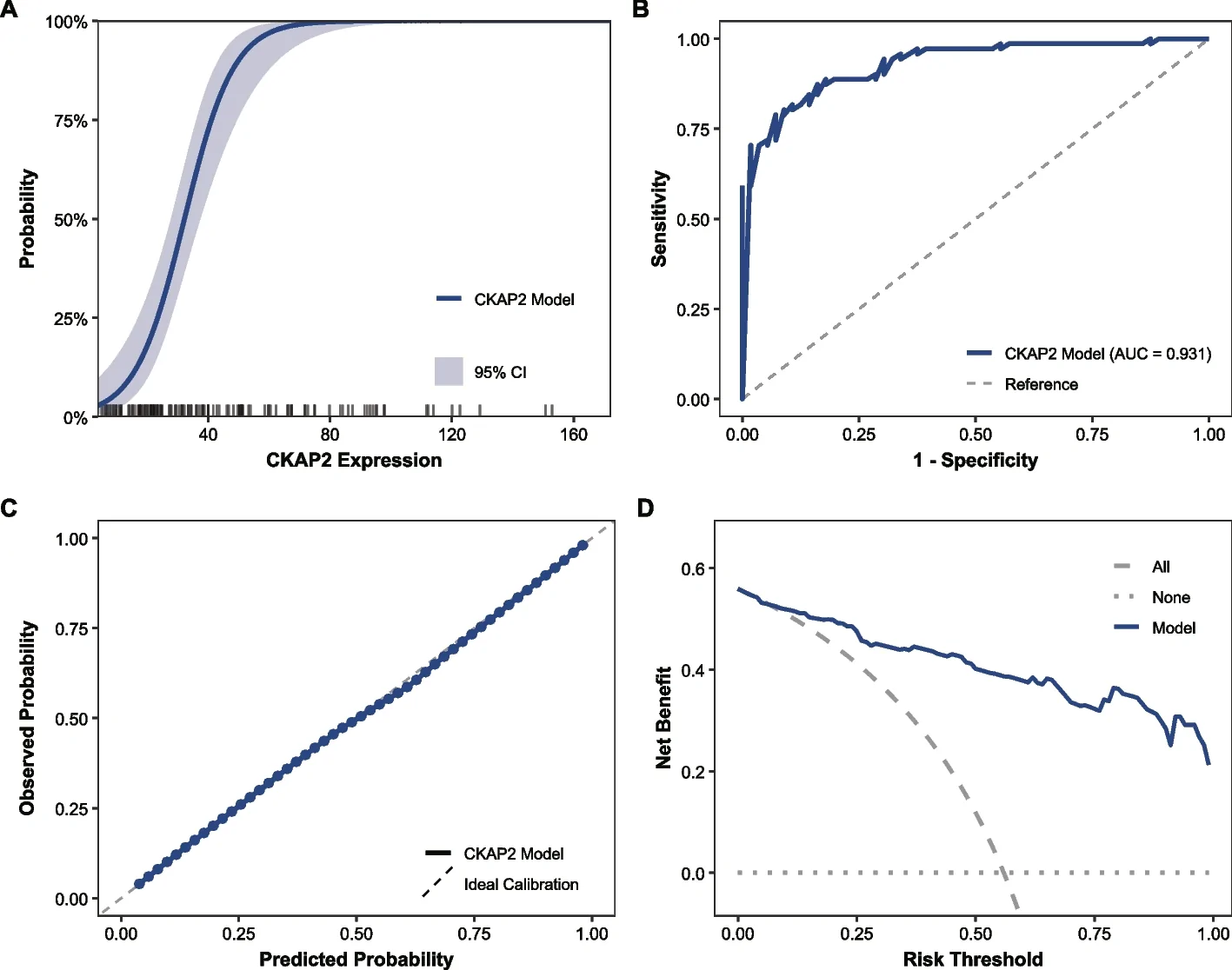
Diagnostic performance and clinical validation of the CKAP2-based logistic regression model in the independent validation cohort. **A** Predicted probability curve illustrating the non-linear relationship between CKAP2 expression levels (x-axis) and the probability of bladder cancer (y-axis). The solid blue line represents the estimated risk probability, and the light blue shaded area indicates the 95% confidence interval (CI). The rug plot along the x-axis displays the distribution of individual samples. **B** Receiver Operating Characteristic (ROC) curve analysis evaluating the discriminative ability of the CKAP2 model. The model achieved an Area Under the Curve (AUC) of 0.931, indicating excellent diagnostic accuracy in distinguishing tumor tissues from normal tissues. **C** Calibration curve assessing the agreement between predicted probabilities and observed outcomes. The x-axis represents the predicted risk of malignancy, and the y-axis represents the actual observed proportion. The gray dashed diagonal line represents ideal prediction (perfect calibration), while the blue solid line represents the bias-corrected performance of the CKAP2 model based on 1,000 bootstrap resamples. **D** Decision Curve Analysis (DCA) quantifying the clinical utility of the model. The y-axis measures the net benefit. The blue solid line represents the CKAP2 diagnostic model. The gray dashed line represents the strategy of assuming all patients have tumors ("Treat All"), and the gray dotted line represents the strategy of assuming no patients have tumors ("Treat None"). The model demonstrates a superior net benefit across a wide range of threshold probabilities

Univariate logistic regression identified CKAP2 as a robust predictor of bladder cancer risk. Elevated CKAP2 levels were significantly associated with an increased probability of malignancy, with an odds ratio of 1.132 (95% CI: 1.082–1.185, P < 0.001) for every unit increase in expression. This non-linear risk trajectory was visualized using a predicted probability curve (Fig. 6A), which illustrated a precipitous rise in tumor probability as CKAP2 levels increased.

The model demonstrated exceptional discriminative performance, achieving an Area Under the Curve (AUC) of 0.931 (95% CI: 0.890–0.973) in the ROC analysis (Fig. 6B). The calibration curve (Fig. 6C) showed high concordance between the predicted risk and actual observed outcomes, with the bias-corrected line closely tracking the ideal diagonal. Furthermore, Decision Curve Analysis (DCA) confirmed the clinical utility of the model, indicating that using CKAP2 for diagnosis provided a substantial net benefit over "treat-all" or "treat-none" strategies across a wide range of threshold probabilities (Fig. 6D). Collectively, these results establish CKAP2 as a high-precision, clinically actionable biomarker.

## Discussion

Bladder cancer remains a prevalent and aggressive malignancy characterized by high recurrence rates, yet the scarcity of sensitive biomarkers continues to impede early detection and effective prognostic stratification. The challenges in bladder cancer management are further compounded by the intricate molecular mechanisms that drive therapeutic resistance. Genetic mutations in genes such as FGFR3, TP53, and RB1, coupled with dysregulation of the PI3K/AKT/mTOR and RAS-MAPK signaling pathways, contribute substantially to both tumorigenesis and the development of resistance to cisplatin-based chemotherapy [31, 32]. Moreover, the tumor microenvironment actively reshapes the immune landscape [33]. Cancer-associated fibroblasts and extracellular matrix remodeling foster an immunosuppressive niche that excludes effector T cells and attenuates responses to immune checkpoint blockade. Within this milieu, tumor-educated cells acquire immunosuppressive phenotypes that further promote tumor progression and immunotherapy resistance [34]. The emergence of intratumoral microbiomes as modulators of immune evasion and genomic instability adds yet another layer of complexity to bladder cancer biology [35]. Given these multifaceted resistance mechanisms, there is an urgent need for reliable biomarkers that can facilitate early detection and guide therapeutic decision-making.

In this study, we integrated multi-omics, Mendelian randomization, and clinical validation to establish CKAP2 as an important diagnostic target. Through differential expression analysis and machine learning selection, we initially identified CKAP2 as a key candidate. This finding was subsequently confirmed in our independent clinical cohort of 127 tissue specimens, where CKAP2 expression was markedly upregulated in tumor tissues. To translate this discovery into clinical practice, we developed a diagnostic model. Visual inspection of the predicted probability curve revealed a distinct saturation effect. The risk of malignancy escalated precipitously when CKAP2 expression exceeded a specific threshold, effectively functioning as a binary switch for tumor identification. This single-biomarker model achieved an exceptional AUC of 0.931, outperforming many conventional multi-gene panels. The performance, supported by calibration curves and decision curve analysis, indicates that CKAP2 provides a cost-effective and clinically practicable tool for establishing a bladder cancer diagnosis with high confidence. Unlike previous studies that relied predominantly on public databases, our work provides compelling evidence from a real-world cohort.

Beyond observational associations, we employed Mendelian randomization to explore the causal relationship between CKAP2 and bladder cancer risk. Observational studies are inherently susceptible to confounding factors. In contrast, Mendelian randomization utilizes genetic variants as unconfounded instrumental variables. Our analysis revealed a significant causal link (OR = 1.103), providing genetic evidence that elevated CKAP2 functions as a driver rather than a mere passenger in bladder carcinogenesis. This causal inference aligns with our functional enrichment analyses. Gene Ontology and KEGG pathway analyses consistently implicated CKAP2 in chromosome segregation, DNA replication, and cell cycle phase transitions. These processes are fundamental to maintaining genomic integrity. Their dysregulation fuels the uncontrolled proliferation characteristic of aggressive bladder tumors [34, 36]. Furthermore, our immunohistochemical analyses and in vitro functional experiments provided direct experimental support for these bioinformatic predictions. CKAP2 knockdown significantly attenuated bladder cancer cell proliferation and migration. These cellular assays reinforce the conclusion that CKAP2 actively contributes to bladder cancer progression rather than serving solely as a passive biomarker. Notably, despite the global upregulation of CKAP2 in tumor tissues, we observed that CKAP2 expression decreases with advancing tumor stage. We hypothesize that this stage-dependent decline is linked to the evolving functional status of p53 during bladder carcinogenesis. Previous studies have established CKAP2 as a p53 target gene and described a positive feedback loop in which CKAP2 activates the G1 tetraploidy checkpoint to prevent aneuploidy in a p53-dependent manner [37]. In early-stage tumors, wild-type p53 likely drives CKAP2 expression as part of a tumor-suppressive mechanism. As bladder cancer progresses, however, TP53 mutations become highly prevalent—up to 50% of invasive tumors harbor TP53 mutations and approximately 76% exhibit loss of p53 function [38]. This p53 dysfunction disrupts the CKAP2-p53 regulatory axis, leading to diminished CKAP2 transcription. Concomitantly, advanced tumors may engage alternative oncogenic pathways, such as FGFR3 mutations, to sustain chromosomal instability and cell cycle progression, thereby reducing reliance on CKAP2 [39]. This dynamic expression pattern not only reconciles the seemingly paradoxical decline in CKAP2 with tumor progression but also may reinforce the value of CKAP2 as a biomarker particularly suited for early-stage detection.

Beyond intrinsic proliferative signaling, CKAP2 may also intersect with pathways governing genomic stability and therapeutic vulnerability. We integrated single-cell RNA sequencing data and observed that CKAP2 expression was predominantly localized to the cancer cell population. This pattern distinguishes CKAP2 from stromal markers. Nevertheless, CellChat analysis demonstrated that CKAP2-high cancer cells are not isolated entities. They actively communicate with stromal components, particularly fibroblasts and macrophages. This tumor-stroma crosstalk is pivotal for remodeling the tumor microenvironment. The inferred interactions suggest that CKAP2-driven tumors may actively suppress anti-tumor immunity by modulating macrophage polarization or promoting T-cell exclusion. These processes facilitate immune evasion and metastasis. Thus, CKAP2 appears to serve as an important node linking intrinsic proliferative signaling with extrinsic microenvironmental modulation.

Further expanding the functional implications of CKAP2, we examined its relationship with the mismatch repair system. Mismatch repair deficiency and microsatellite instability-high status have been established as predictive markers for sensitivity to immune checkpoint inhibition across multiple cancer types, including a subset of urothelial carcinomas. However, the prevalence of dMMR/MSI-H in bladder cancer is relatively low, with pooled estimates of approximately 2.30% for dMMR and 2.11% for MSI-H in bladder cancer specifically [40]. Importantly, discordance between MMR protein expression and molecular MSI status has been documented, particularly in non-colorectal malignancies, raising questions about the interchangeability of these predictive markers. Given this established connection between mismatch repair deficiency and microsatellite instability, we analyzed the expression of four core MMR genes—MLH1, MSH2, MSH6, and PMS2—in our internal bladder cancer cohort stratified by CKAP2 expression. Notably, all four MMR genes exhibited significantly higher expression in the CKAP2-high group compared with the CKAP2-low group. Spearman correlation analysis further confirmed positive correlations between CKAP2 and each MMR gene. These findings suggest that CKAP2 overexpression is associated with transcriptional upregulation of the MMR machinery in bladder cancer. This pattern may reflect a compensatory response to heightened replication stress or may be linked to the cell cycle-dependent expression of both CKAP2 and MMR components. Nevertheless, the clinical implications of this association remain to be fully elucidated. Future studies should determine whether CKAP2-high tumors exhibit distinct sensitivity or resistance profiles to immunotherapeutic agents.

Despite the comprehensive nature of this study, several limitations warrant consideration. First, although the diagnostic model was validated in an independent cohort, the study design remains retrospective. Large-scale prospective multicenter trials are necessary to further establish the clinical utility of the CKAP2-based diagnostic model in routine practice. Second, with respect to the Mendelian randomization analysis, the expression quantitative trait loci (eQTLs) employed as instrumental variables were derived exclusively from whole blood samples obtained from the eQTLGen Consortium, as bladder tissue-specific eQTL data were unavailable. Given the inherent tissue-specificity of gene expression regulation, blood-derived eQTLs may not fully capture the true regulatory dynamics within bladder tissue. This discrepancy could potentially introduce bias in the estimation of causal effects. Future validation utilizing bladder tissue-specific eQTL resources, should such data become accessible, will be essential to corroborate our causal inferences. Third, while our immunohistochemical analyses and cellular functional assays demonstrated that CKAP2 promotes bladder cancer cell proliferation and migration, these experiments represent only an initial characterization of its functional role. More detailed in vivo experiments and mechanism analyses are needed. Additionally, comprehensive molecular studies are needed to delineate the precise signaling cascades through which CKAP2 influences cell cycle progression and tumor-stroma communication. Fourth, the single-cell RNA-seq analysis was performed using the GSE145137 dataset, which only includes samples from one bladder cancer patient and patient-derived xenograft (PDX) samples, resulting in an extremely small sample size. Moreover, single-cell transcriptomic data from the clinical cohort of this study were not generated to provide independent support. As a consequence, the generalizability of the tumor-stroma crosstalk results inferred from CellChat must be interpreted with caution. Future studies incorporating single-cell data from larger, multi-patient cohorts or from our own well-characterized clinical samples will be essential to validate these microenvironment-related findings and strengthen their translational relevance. Fifth, the observed association between CKAP2 and MMR genes expression raise intriguing questions about potential therapeutic implications. Future research should examine whether CKAP2 expression levels correlate with response to immune checkpoint inhibitors or DNA-damaging chemotherapeutic agents [31]. Such studies could reveal whether CKAP2 possesses utility not only as a diagnostic biomarker but also as a predictive marker for guiding treatment selection.

## Conclusion

In conclusion, by integrating multi-omics mining, machine learning, and Mendelian randomization, this study systematically identified and characterized CKAP2 as an important driver of bladder cancer. Beyond bioinformatic predictions, our analyses elucidate that CKAP2 promotes tumorigenesis through intrinsic cell cycle dysregulation and extrinsic remodeling of the tumor microenvironment via cancer-fibroblast crosstalk. Crucially, the validation in an independent clinical cohort of 127 tissue specimens distinguishes this work, establishing a highly accurate diagnostic model with an AUC of 0.931. These results confirm CKAP2 as a robust, clinically actionable biomarker, providing a solid foundation for improved early detection and personalized management of bladder cancer.

## Supplementary Information

Supplementary Material 1: Fig S1. Association between CKAP2 expression and clinical characteristics in the internal cohort.

Supplementary Material 2: Fig S2. GO enrichment analysis of CKAP2 high- and low-expression groups in the internal and TCGA cohorts.

Supplementary Material 3: Fig S3. KEGG enrichment analysis of CKAP2 high- and low-expression groups in the internal and TCGA cohorts.

Supplementary Material 4: Fig S4. MMR-related genes expression in CKAP2 high- and low-expression groups in the internal cohort.

Supplementary Material 5: Fig S5. Kaplan–Meier survival curves comparing high and low CKAP2 expression groups in the internal cohort

Supplementary Material 6. Data. The corresponding data IDs.

## Data Availability

The exposure data used for Mendelian randomization analyses are publicly available from the IEU OpenGWAS database (https://gwas.mrcieu.ac.uk/). The outcome summary statistics are accessible via the Finnish database (https://www.finngen.fi/en). Bladder cancer transcriptomic data can be obtained from The Cancer Genome Atlas (TCGA) (https://www.cancer.gov/tcga) and the transcriptome (bulk RNA-seq data) analyzed in this study was obtained from the HRA005001 and HRA003461 datasets (https://ngdc.cncb.ac.cn), and the single-cell RNA sequencing data for bladder cancer are available from the Gene Expression Omnibus (GEO) database (https://www.ncbi.nlm.nih.gov/geo/)：GSE145137.
