# Supplementary material for: Multi-omics integration and clinical validation identify CKAP2 as a diagnostic biomarker for bladder cancer
Source: World J Surg Oncol. 2026 Jun 23;24:358. doi: 10.1186/s12957-026-04464-7 (PMC13543375; doi:10.1186/s12957-026-04464-7)
Supplement: Supplementary file 1 [file 12957_2026_4464_MOESM1_ESM.pdf]

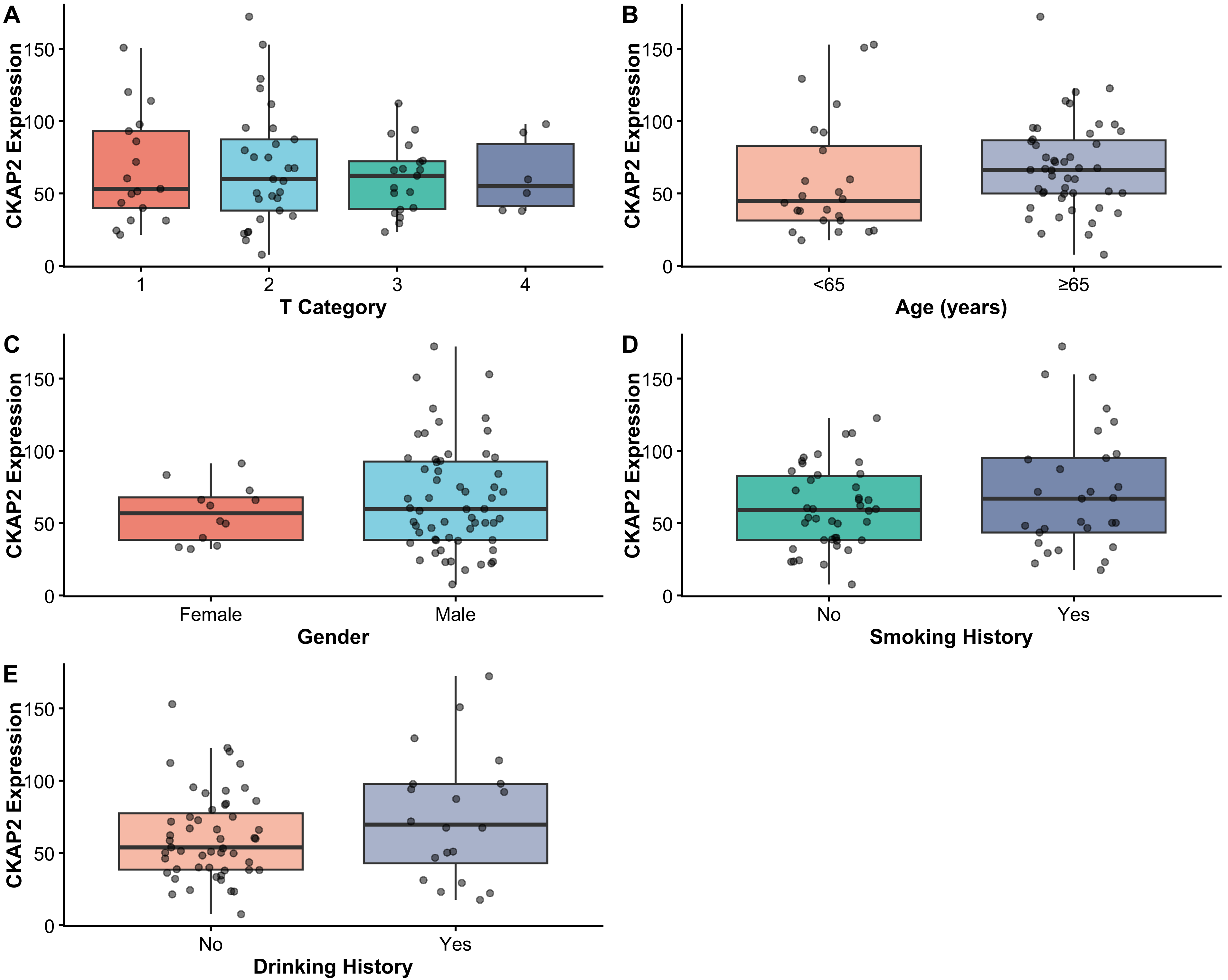

A
B
C
D
E
CKAP2 Expression
0
50
100
150
T Category
1
2
3
4
Age (years)
<65
≥65
Gender
Female
Male
Smoking History
No
Yes
Drinking History
No
Yes
