# Supplementary material for: Multi-omics integration and clinical validation identify CKAP2 as a diagnostic biomarker for bladder cancer
Source: World J Surg Oncol. 2026 Jun 23;24:358. doi: 10.1186/s12957-026-04464-7 (PMC13543375; doi:10.1186/s12957-026-04464-7)
Supplement: Supplementary file 2 [file 12957_2026_4464_MOESM2_ESM.pdf]

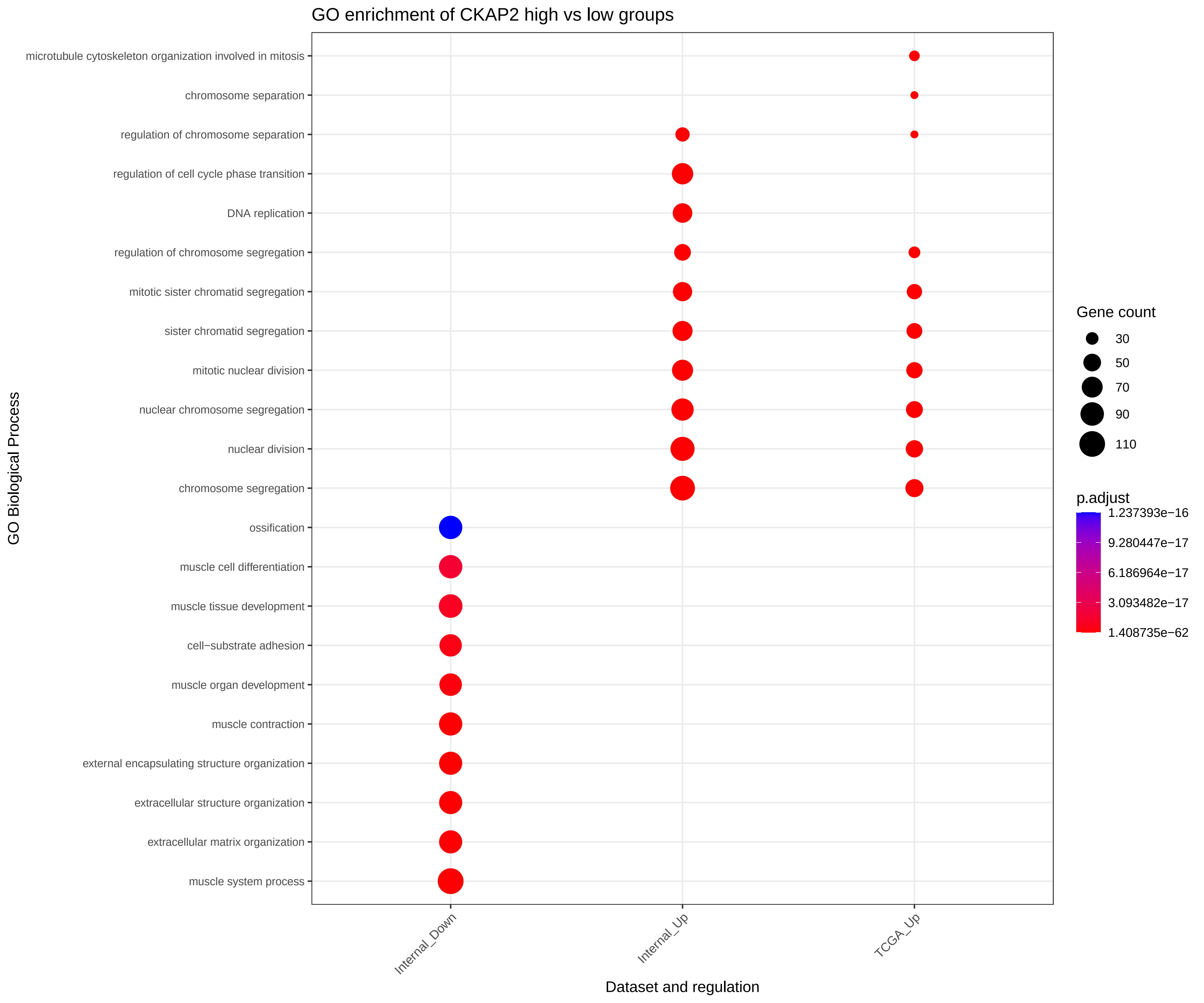
GO enrichment of CKAP2 high vs low groups
microtubule cytoskeleton organization involved in mitosis
chromosome separation
regulation of chromosome separation
regulation of cell cycle phase transition
DNA replication
regulation of chromosome segregation
mitotic sister chromatid segregation
sister chromatid segregation
mitotic nuclear division
nuclear chromosome segregation
nuclear division
chromosome segregation
ossification
muscle cell differentiation
muscle tissue development
cell−substrate adhesion
muscle organ development
muscle contraction
external encapsulating structure organization
extracellular structure organization
extracellular matrix organization
muscle system process
GO Biological Process
Internal_Down
Internal_Up
TCGA_Up
Dataset and regulation
Gene count
30
50
70
90
110
p.adjust
1.237393e−16
9.280447e−17
6.186964e−17
3.093482e−17
1.408735e−62
