# Supplementary material for: Multi-omics integration and clinical validation identify CKAP2 as a diagnostic biomarker for bladder cancer
Source: World J Surg Oncol. 2026 Jun 23;24:358. doi: 10.1186/s12957-026-04464-7 (PMC13543375; doi:10.1186/s12957-026-04464-7)
Supplement: Supplementary file 3 [file 12957_2026_4464_MOESM3_ESM.pdf]

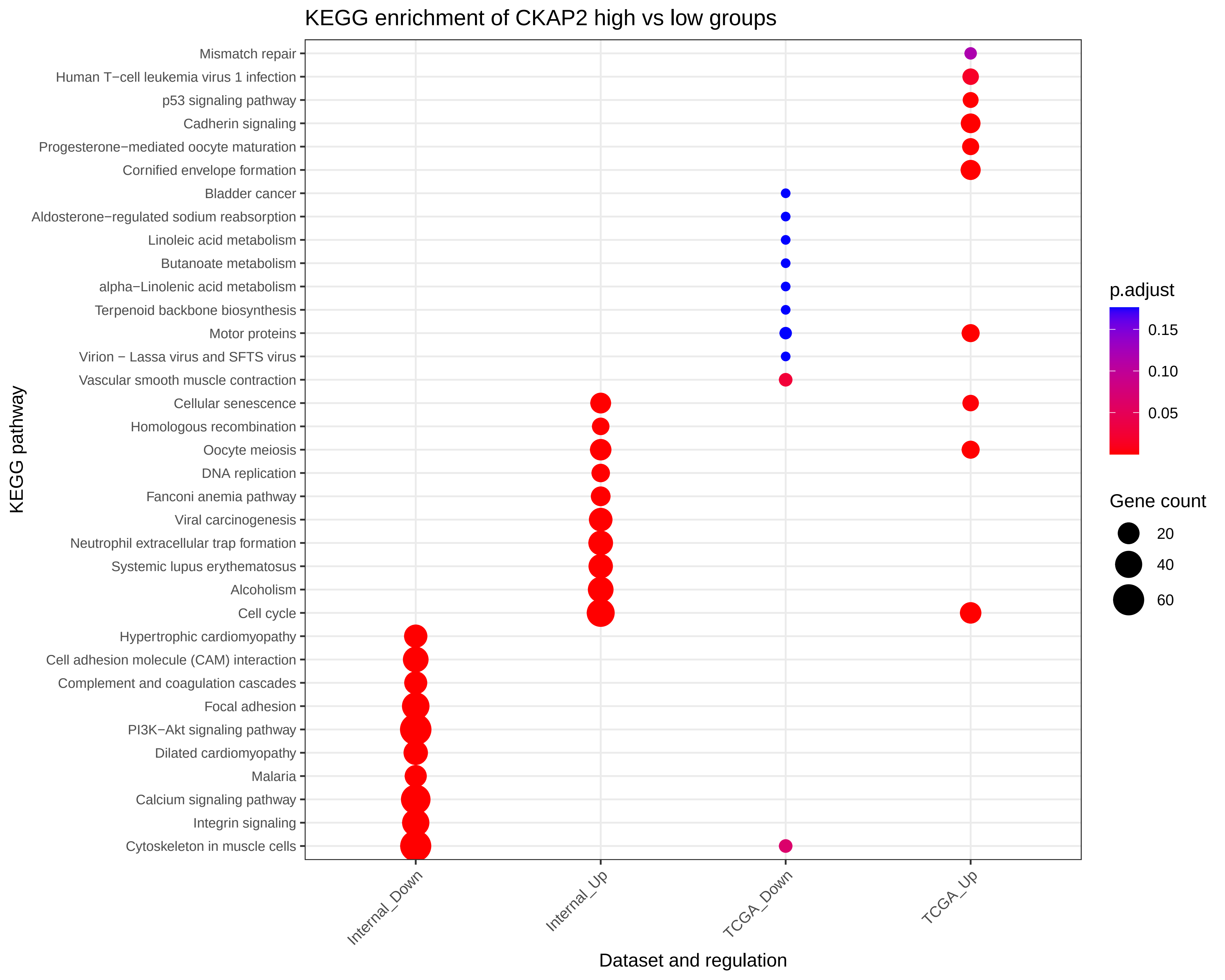

KEGG enrichment of CKAP2 high vs low groups
Mismatch repair
Human T−cell leukemia virus 1 infection
p53 signaling pathway
Cadherin signaling
Progesterone−mediated oocyte maturation
Cornified envelope formation
Bladder cancer
Aldosterone−regulated sodium reabsorption
Linoleic acid metabolism
Butanoate metabolism
alpha−Linolenic acid metabolism
Terpenoid backbone biosynthesis
Motor proteins
Virion − Lassa virus and SFTS virus
Vascular smooth muscle contraction
Cellular senescence
Homologous recombination
Oocyte meiosis
DNA replication
Fanconi anemia pathway
Viral carcinogenesis
Neutrophil extracellular trap formation
Systemic lupus erythematosus
Alcoholism
Cell cycle
Hypertrophic cardiomyopathy
Cell adhesion molecule (CAM) interaction
Complement and coagulation cascades
Focal adhesion
PI3K−Akt signaling pathway
Dilated cardiomyopathy
Malaria
Calcium signaling pathway
Integrin signaling
Cytoskeleton in muscle cells
KEGG pathway
Internal_Down
Internal_Up
TCGA_Down
TCGA_Up
Dataset and regulation
p.adjust
0.15
0.10
0.05
Gene count
20
40
60
