# Supplementary material for: Multi-omics integration and clinical validation identify CKAP2 as a diagnostic biomarker for bladder cancer
Source: World J Surg Oncol. 2026 Jun 23;24:358. doi: 10.1186/s12957-026-04464-7 (PMC13543375; doi:10.1186/s12957-026-04464-7)
Supplement: Supplementary file 4 [file 12957_2026_4464_MOESM4_ESM.pdf]

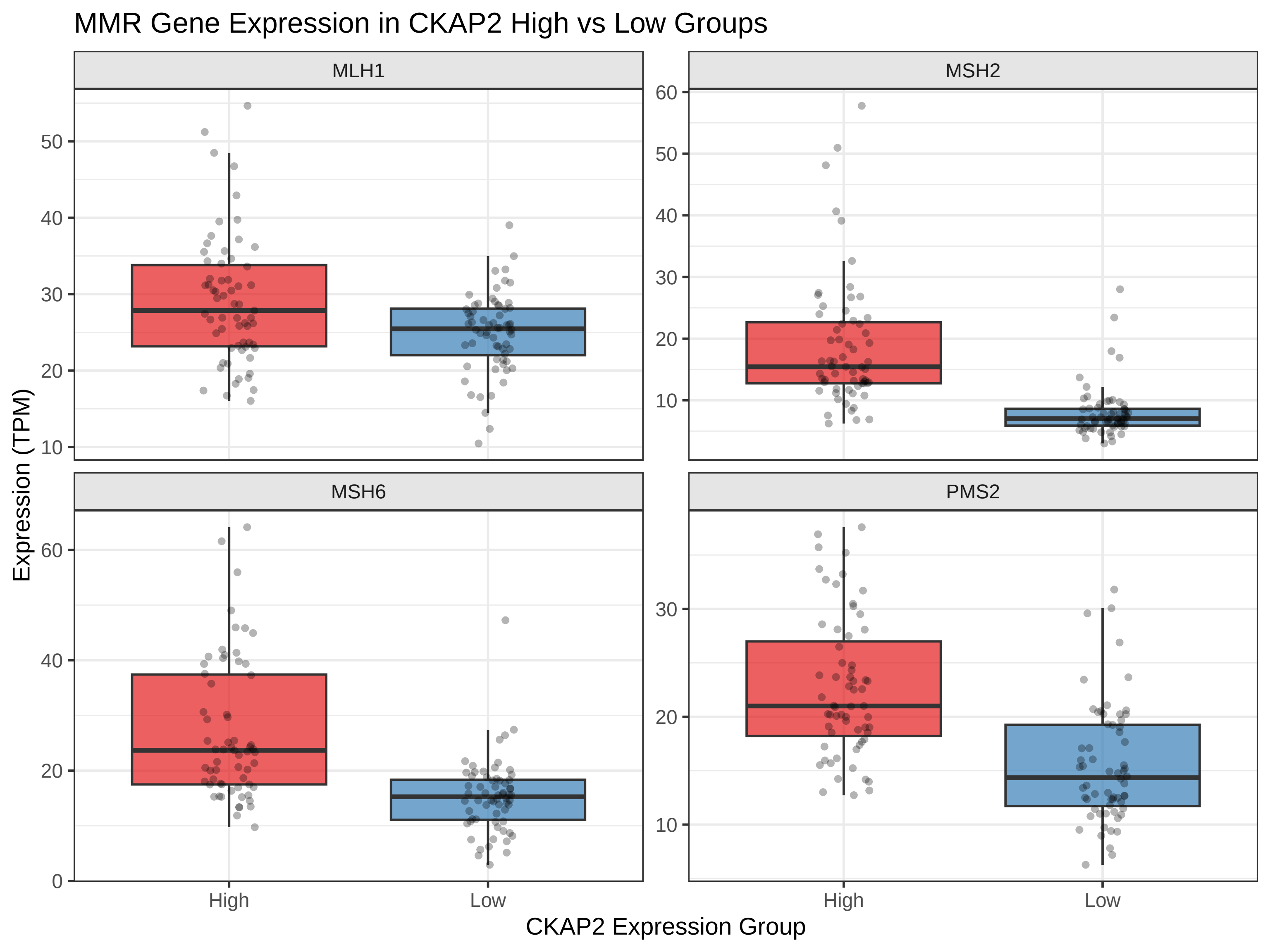
MMR Gene Expression in CKAP2 High vs Low Groups
MLH1
MSH2
MSH6
PMS2
Expression (TPM)
CKAP2 Expression Group
High
Low
10
20
30
40
50
60
0
