# Supplementary material for: Multi-omics integration and clinical validation identify CKAP2 as a diagnostic biomarker for bladder cancer
Source: World J Surg Oncol. 2026 Jun 23;24:358. doi: 10.1186/s12957-026-04464-7 (PMC13543375; doi:10.1186/s12957-026-04464-7)
Supplement: Supplementary file 5 [file 12957_2026_4464_MOESM5_ESM.pdf]

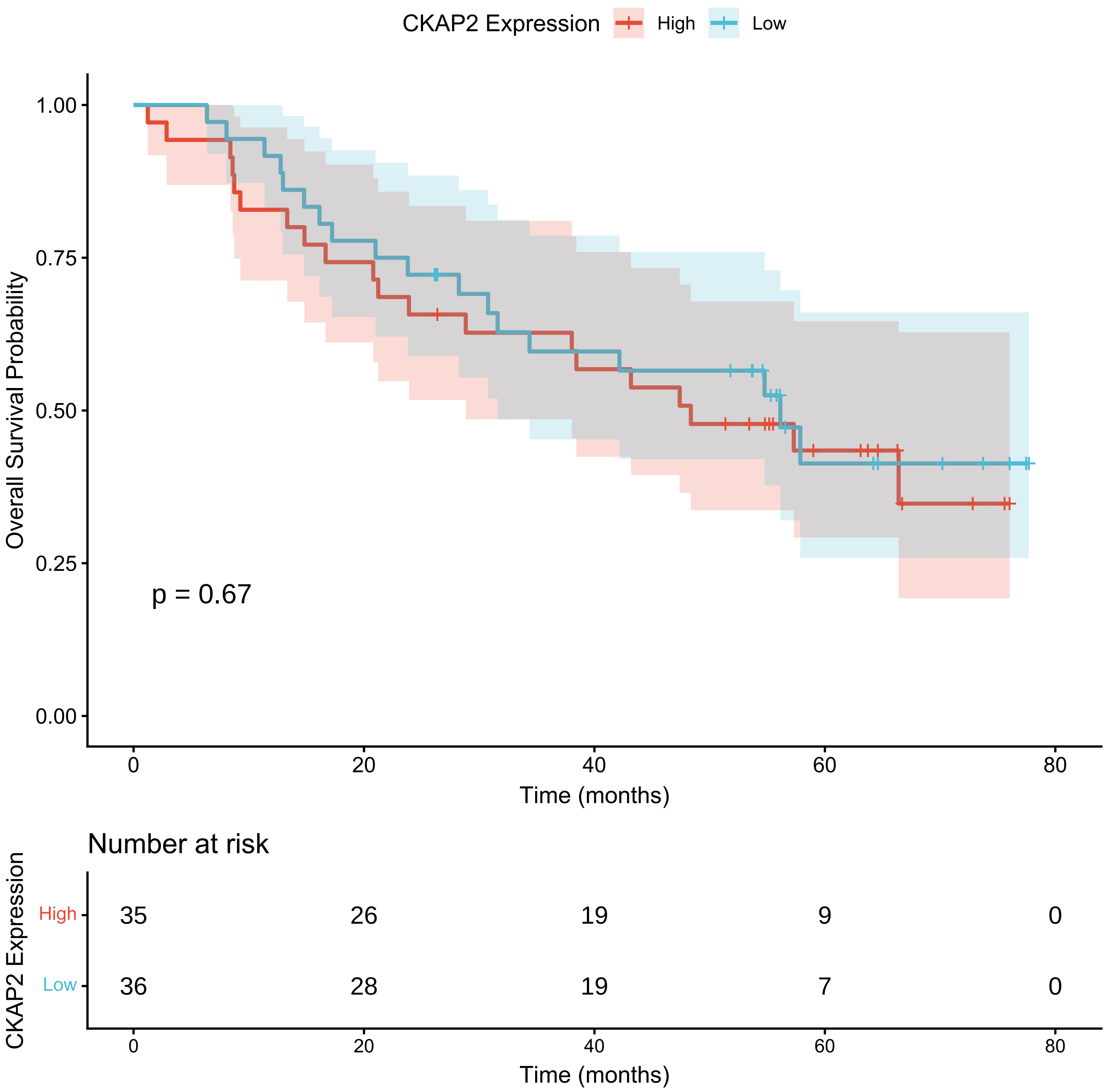

CKAP2 Expression
High
Low
1.00
0.75
0.50
0.25
0.00
Overall Survival Probability
p = 0.67
0
20
40
60
80
Time (months)
Number at risk
CKAP2 Expression
High 35 26 19 9 0
Low 36 28 19 7 0
0
20
40
60
80
Time (months)
